# A practical approach to the nutritional management of chronic kidney disease patients in Cape Town, South Africa

**DOI:** 10.1186/s12882-016-0297-4

**Published:** 2016-07-08

**Authors:** Oluwatoyin I. Ameh, Lynette Cilliers, Ikechi G. Okpechi

**Affiliations:** Division of Nephrology and Hypertension, Groote Schuur Hospital and University of Cape Town, Cape Town, South Africa; Department of Dietetics, Groote Schuur Hospital, Cape Town, South Africa; Division of Nephrology and Hypertension, E13 Renal Unit Groote Schuur Hospital and University of Cape Town, Observatory, 7925 Cape Town, South Africa

**Keywords:** Protein, Potassium, Phosphate, Restriction, Nutrition, Kidney disease

## Abstract

**Background:**

The multi-racial and multi-ethnic population of South Africa has significant variation in their nutritional habits with many black South Africans undergoing a nutritional transition to Western type diets. In this review, we describe our practical approaches to the dietary and nutritional management of chronic kidney disease (CKD) patients in Cape Town, South Africa.

**Discussion:**

Due to poverty and socio-economic constraints, significant challenges still exist with regard to achieving the nutritional needs and adequate dietary counselling of many CKD patients (pre-dialysis and dialysis) in South Africa. Inadequate workforce to meet the educational and counselling needs of patients, inability of many patients to effectively come to terms with changing body and metabolic needs due to ongoing kidney disease, issues of adherence to fluid and food restrictions as well as adherence to medications and in some cases the inability to obtain adequate daily food supplies make up some of these challenges. A multi-disciplinary approach (dietitians, nurses and nephrologists) of regularly reminding and educating patients on dietary (especially low protein diets) and nutritional needs is practiced. The South African Renal exchange list consisting of groups of food items with the same nutritional content has been developed as a practical tool to be used by dietitians to convert individualized nutritional prescriptions into meal plan to meet the nutritional needs of patients in South Africa. The list is currently utilized in counselling CKD patients and provides varied options for food items within the same group (exchangeable) as well as offering ease for the description of suitable meal portions (sizes) to our patients.

**Summary:**

Regular and continuous education of CKD patients by a multi-disciplinary team in South Africa enables our patients to meet their nutritional goals and retard CKD progression. The South African renal exchange list has proved to be a very useful tool in meeting this need.

**Electronic supplementary material:**

The online version of this article (doi:10.1186/s12882-016-0297-4) contains supplementary material, which is available to authorized users.

## Background

Chronic kidney disease (CKD) continues to impose an increased burden on scarce health resources in sub-Saharan Africa (SSA) where there is a transition in epidemiology from the predominance of communicable diseases to the co-dominance of communicable and non-communicable diseases [1, 2]. The exact epidemiologic dimensions of CKD in SSA remain largely unknown due to the unavailability of national renal registries. Available data however indicates a pooled prevalence of 13.9 % for the entire region [3]. In South Africa, prevalence was 14.3 %, in Kenya 4.0 % and 8.0 % in Sudan. Rural and urban dwellers in the region have also been noted to have similar CKD prevalence rates [3].

CKD represents a state of altered metabolism associated with a variety of nutritional deficiencies and requirements along the entire spectrum of CKD. In addition to variation across the stages of CKD, these nutritional deficiencies and requirements differ according to the cause of CKD, genetic and environmental factors as well as the individual’s unique metabolic type [4]. Among end-stage renal disease (ESRD) patients on dialysis, nutritional deficiencies are known to also vary according to dialysis modality. Protein-energy wasting is common in CKD patients with causes including decreased intake, concurrent illnesses, increased catabolism (especially in haemodialysis patients), losses of nutrients into dialysate (particularly amino acids, peptides and protein in CAPD) and diagnostic or therapeutic procedures that reduce nutrient intake or stimulate net protein breakdown (e.g corticosteroids) [5].

Protein-energy wasting, sodium and water retention, vitamin D deficiency, and phosphate retention are some of the nutritional abnormalities commonly encountered in CKD patients. Others include potassium retention, loss of water and fat soluble vitamins, and mineral and trace elements deficiencies. While the management of the nutritional complications of CKD requires an understanding of the pathophysiologic processes at play, equally as important, is an understanding of the dietary peculiarities of various regions/populations. This guarantees a tailored approach to the management of the nutritional aspects of CKD in various regions.

South Africa’s population is multi-racial and multi-ethnic and therefore there is a significant variation in the nutritional habits of the different constituent groups. White South Africans have largely retained the typical western diet of refined starch, animal protein and fats while the diet of black South Africans’ is predominantly carbohydrate based [6]. The dietary practices of Coloured and Indian South Africans has been influenced by religion and tradition with pork and non-halaal meats prohibited among Muslims while vegetarianism is practiced among some Hindu Indians [6]. A nutritional transition has been observed predominantly between urban and rural dwelling blacks. Carbohydrates comprise approximately 60 % of total energy intake of urban dwelling blacks while more than 50 % of protein intake is derived from animal protein; conversely among rural dwelling blacks, plant protein accounts for the greater percentage of total protein intake while up to 80 % of total energy intake is carbohydrate. Urban dwelling blacks also have a higher intake of fats in comparison to their rural dwelling counterparts - approximately 25 % total fat intake versus 18 % total fat intake respectively [6]. The aim of this review is to present the challenges we encounter in prescribing low protein diets and other nutritional management of CKD patients in South Africa and to highlight some of the practical approaches utilized in this process.

### Challenges and peculiarities of dietary interventions in CKD in South Africa

Socioeconomic inequality in South Africa is substantial with a high *Gini coefficient* (an index used to measure income distribution with higher scores indicating more inequality) [7, 8]. A strategy in offsetting this inequality in the South African health sector includes the provision of healthcare access for the indigent at government-run public hospitals. As a result of this, CKD patients managed in such settings are usually indigent with no health insurance and commonly a low level of education. This often impacts on, for example, understanding why diets should change or be restricted in CKD. The poor socioeconomic status of patients who access public health services also negatively affects purchasing power and by extension, limits total adherence to prescribed nutritional modifications. A high unemployment rate and often lack of a stable income means an inability to afford all meals and therefore a dependence on food parcels provided by non-profit organizations such as the Cape Kidney Association. CKD patients with household food insecurity are given parcels of non-perishable food items (worth about USD $20) once every 2 weeks until a social grant from the government is arranged. Contents of the parcel are as dictated by social welfare. Items in the parcel can feed an individual for approximately 3 weeks and a small family of 3–4 people for 1 week.

In addition to socioeconomic inequity, there is a shortage of health care workers in all specialties in South Africa and indeed Africa [9, 10]. For instance, at our centre, a 900-bed hospital, there are only 10 dietitians responsible for the care of all inpatients and assessment of patients referred from various out-patient clinics; responsibilities which should ideally be undertaken by at least 19 dietitians. The amount of dedicated care/counselling needed with CKD patients often becomes challenging in many instances and thus limits the frequency of dietary assessments of patients. Renal dietetics has also not fully developed in South Africa and many SSA countries. Even where dietitians may be available, a thorough knowledge of prescription of low protein diets or other dietary and nutritional education and counselling of patients may not be firmly established given that there are not a lot of research on diets and CKD in Africa. One study designed to assess the practices of South African dietitians regarding the dietary treatment of patients with CKD reported a low level of exposure of dietitians to renal patients (28 %) with substantial deviation from international standards reported in their prescriptions [11].

### General approaches to the nutritional management of CKD

Many patients feel overwhelmed in the initial phase of the diagnosis of CKD and the attendant dietary alterations required are daunting especially in situations where understanding the basis of such alterations are lacking. The alterations to the diets of patients with CKD differ on an individual basis (usually taking into consideration co-morbidities and individual body composition); however certain elements remain applicable to all patients.

Given that several patients in our setting present to us for the first time when they require dialysis, the dietary and nutritional management of CKD patients often start quite late. In many instances, you want to wait and allow the patient to get used to dialysis therapy and fluid restrictions. The process of dietary counselling often starts with a simple educational session highlighting the need to alter diet in the context of CKD, the function of different nutrients in the body, the dangers of excesses or deficiencies of certain nutrients, the foods that are allowed, those that should be restricted, and the support structure available to practically implement these changes in everyday living. For non-English or non-Afrikaans speaking patients, interpreters help in translating these concepts into the patient’s best understood language. Also, the dietary/nutritional education is usually targeted at the household of the CKD patient (especially those whose roles involve the purchase and preparation of meals, for example, mothers or wives) rather than the individual patient. We have found this approach to be more effective in bringing about and maintaining, the desired change in dietary habits.

### Approaches to low protein diets

Daily dietary protein allowance among CKD patients varies according to the stage of CKD, associated co-morbidities, and the modality of renal replacement [12–14]. Generally, we follow standard guidelines for our prescriptions given that we do not have our own data to guide us [13, 15]. Hence, we restrict protein intake to 0.6–0.8 g/kg ideal body weight (IBW) for pre-dialysis patients and prescribe 1.2 g/kg IBW for hemodialysis patients and 1.2 - 1.3 g/kg ideal body weight for peritoneal dialysis patients [16].

**Fig. 1:**
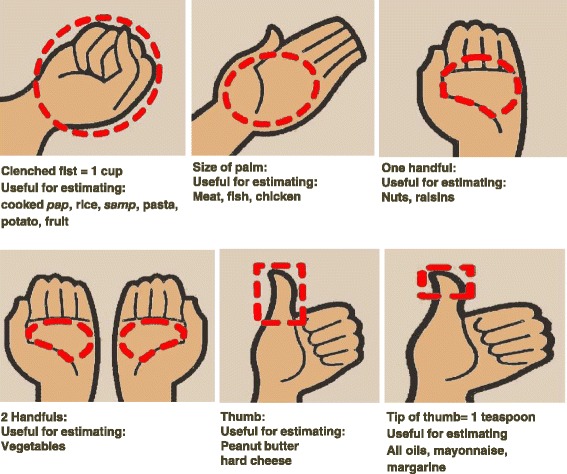
Using your hand as a guide to estimate portion size

The successful implementation of low protein diets in our patients rests on the correct proportioning of all food groups in a healthy and balanced diet. Table 1 shows an example of a low protein diet meal-portions used for portioning meal sizes and frequency per day in pre-dialysis and dialysis CKD patients in our centre in order to limit protein intake. A practical tool utilized when describing portion sizes to some of our patients (in addition to comparison to household items) is the approximation of volumes from hand measurements (Fig. 1). For example, the tip of the finger is equivalent to 1 teaspoon, the palm equivalent to 90 g (3 matchboxes) of meat, chicken, or fish, and the fist equivalent to 1 cup as well as to 1 portion of fruits. Other practical measures include using smaller plates and serving utensils, avoiding second helpings, eating at a table rather than in front of a television, sticking to regular meal and snack times and keeping healthier food options within easy reach. Table 2 shows practical examples of a daily low protein diet meal-plan utilized for pre-dialysis and dialysis CKD patients in our centre.

**Table 1:** Example menu for low protein diet meal-portions used for pre-dialysis and a dialysis patients in Cape Town^a^

Exchanges	Recommended daily portions	
	Pre-Dialysis	Dialysis
Meat – Low phosphate	2	5
Meat – High phosphate	1	1
Meat - Legumes	1	2
Milk	1	1
Starch	9	8
Vegetable	4	4
Fruit	3	3
Drinks	1	1
Sugar	4	2
Fat	5	3

^a^Exchanges and portions based on the South African Renal Exchange lists

**Table 2:** Example menu for low protein diet meal-plans used for pre-dialysis and dialysis patients in Cape Town^a^

Meal-plan	Pre-Dialysis meal plan	Haemodialysis meal plan	Possible substitute
Breakfast	4 heaped ladle spoon oats	4 heaped ladle spoon oats	4 heaped ladles soft porridge
	125 ml (1/2 cup) milk	125 ml (1/2 cup) milk	125 ml (1/2 cup) yogurt
	1 teaspoon sugar	1 teaspoon sugar	Sweetened yogurt
Snack time	2 slices white bread	1 slice white bread	Bread Rolls (2:1 rolls)/Provita biscuits (6:3 biscuits)
	Egg with 2 teaspoons of mayonnaise	Egg with a teaspoon of mayonnaise	Matchbox size cheese
	Small apple	Small apple	Quarter avocado pear (if potassium requirements allow)
Lunch	3 heaped table spoons rice	3 heaped table spoons rice	Baked or boiled potato (if potassium requirements allow)l
	1 heaped table spoon *Bobotie*	3 heaped table spoon *Bobotie*	Mince, level dessert spoons 3:9
	½ cup mixed vegetables AND 1 medium onion in dish	½ cup mixed vegetables AND medium onion in dish	1 cup of salad
	1 teaspoon oil/2 level teaspoons margarine for cooking	1 teaspoon oil/2 level teaspoons margarine for cooking	1 level teaspoon French salad dressing
Snack time	6 Litchi	6 Litchi	Medium *Naartjie* (if potassium requirements allow)
Supper	2 heaped table spoons cooked pasta	2 heaped table spoons cooked pasta	Medium roti (30 cm diameter)
	Chicken (small drumstick / matchbox size)	Chicken breast (2 x small drumstick/ 2 x matchbox size)	Chicken breast (½:1)
	½ medium cooked union & Heaped table spoon peas & 4 medium slices Green pepper & Garlic gloves	1 big slice tomato Shredded lettuce Slice of onion AND ½ cup coleslaw	¼ cup sweetcorn AND ½ cup mixed green salad
	1 teaspoon oil / 2 level teaspoons margarine for cooking	1 teaspoon oil / 2 level teaspoons margarine for cooking	1 teaspoon oil / 2 level teaspoons margarine included in roti
During the day^b^	125 ml / ½ cup apple juice	125 ml / ½ cup apple juice	125 ml / ½ cup mango juice
	2cups low salt / sugar coated popcorn	2cups low salt / sugar coated popcorn	3 cream cracker With thinly spread jam
	1 medium handful unsalted peanuts	2 medium handful unsalted peanuts	*Biryani* or beans included in a main meal
	Small cup of tea With a teaspoon of sugar / honey	Small cup of tea With a teaspoon of sugar/hone	½ cup *Mageu*
	4 hard boiled or soft jelly sweets	2 hard boiled or soft jelly sweets	Peppermints; (6 sweets: 3 sweets)

^a^The above meal plan is an example developed for an average non-diabetic 70 kg patient

^b^Patients are given the prerogative to add these on to any of the meals during the day

The education and re-education (at clinic or dialysis visits) of our patients on the various perils of injudicious protein intake in CKD have been pivotal elements in maintaining compliance to prescribed daily protein requirements. We educate on the role that unrestricted protein intake plays in the progression of CKD and how protein intake also influences blood urea nitrogen (BUN) levels i.e. urea being the breakdown product of protein metabolism. The relative bioavailability of various proteins in leading to increased BUN and phosphate is also explained. Phosphate control is introduced to the patient while discussing protein allowances as proteins also contain phosphorus [17]. These high biologic value proteins are usually obtained from animal sources and thus also contain substantial amounts of phosphate (organic phosphates) that are relatively more efficiently absorbed in humans [17]. As a result, we also educate our patients about portions that deliver required proteins with the least ability to raise serum phosphate levels. Table 3 features some South African food items, according to their protein and phosphate content and suggested portion sizes for adult dialysis patients.

**Table 3:** South Africa food items by protein and phosphate contents and suggested portion sizes for adult dialysis patients

Low Phosphate Meat and Meat alternatives		High Phosphate Meat, meat alternatives and dairy		Discouraged meat, meat alternatives and dairy	
Item	Portion Size	Item	Portion Size	Avoided Item	Rationale
Beef stew with Vegetables	60 g/¼ cup	Pilchards	30 g/1 Large/2 Small	Fish paste	Very high in sodium
*Bobotie* (regular mince)	40 g/1 heaped tbsp^a^	Cheese	30 g/size of matchbox	Cheese spread or wedges	high in phosphate and sodium
Tuna (canned in oil or brine)	30 g/¼ cup	Egg	1 extra large	Feta	high in phosphate and sodium
Chicken	30 g/1 small drumstick	Liver	30 g/1 Chicken Liver/size of small matchbox	Marmite or Bovril	High in phosphate, sodium and potassium
Chicken stew with vegetables	60 g/¼ cup	Bacon High Na	30 g/3 Rashers	Nuts	high in phosphate and potassium
Cottage cheese	60 g/2 heaped tbsp.	*Biryani*	2 heaped large spoons	*Bokkoms*	Very high in sodium
Cottage pie (regular mince)	50 g/2 heaped dessert spoons	Peanut butter	2 level dessertspoons	Offal	Very high in fat
Fish (snoek, butterfish, herring)	30 g/size of ½ hand palm	Milk	½ cup		
Kidneys (Beef)	30 g/3 heaped tbsp.	Baked beans	2 heaped tablespoons		
Kidneys (mutton)	1 kidney				
Lasagne (lean mince)	75 g				
Meatballs (regular mince)	30 g/1 small meatball				
Mince	30 g/3 level dessertspoons				
Mutton stew with vegetables	60 g/¼ cup				
Patty, beef, grilled	40 g/1 small				
Spaghetti Bolognaise, lean mince	75 g				
Biltong High Na	20 g/6 slices				
Chicken pie High Na	70 g/1 small square				
Corned meat, canned High Na	30 g/2 thin slices				
Polony/cold meat(10cmdiameter) High Na	60 g/4 thin slices				
Salami/Russian (5 cm diameter) High Na	30 g/5 thin slices				
Sausage, Boerewors, thick High Na	60 g/10 cm piece				
Viennas High Na	70 g/2 large				

^a^tbsp. table spoon(s)

A practical challenge encountered in the prescription of dietary protein to our patients, especially high biologic proteins like milk, eggs, meat, and chicken, is the inability of patients to afford these items on a long term basis due to poor socioeconomic status. A similar challenge is seen also among patients who depend on food parcels. We therefore encourage alternatively low cost, lower biologic value proteins such as legumes in combination with portions of high biologic proteins as can be afforded by each patient. While this approach has the advantage of limiting high phosphate derived from proteins (plant phosphate less efficiently absorbed), it also has the potential disadvantage of not providing all required essential amino acids to our patients. We are able to supplement the amino acid requirements of our dialysis patients that are at high risk of protein malnutrition with enteral nutritional supplements such as protein-energy sip feeds (Fresubin®) which contains essential and non-essential amino acids. Dialysis patients receive 250 ml of Fresubin® on alternate days at dialysis sessions; however, this is not available to pre-dialysis patients who are given nutritional supplements in the form of a fortified porridge provided on a monthly basis as take home stock.

Additionally, we utilize the South African Renal exchange (SARE) list which consists of groups of food items with the same nutritional content (energy, protein, fat, potassium, sodium and phosphates) [18]. Food items within the same group are considered exchangeable, thus promoting varied food options with the same nutritional and caloric content. A further characteristic of the SARE list is the translation of portion sizes of items in the list to readily understandable measures (Fig. 1). A good understanding of the concepts of protein intake and CKD progression, protein types, constituents and portion sizes by our pre-dialysis patients is especially important in our resource-constrained setting where dialysis rationing is operational in the public health sector.

### Dietary approaches to phosphate control

It is often a struggle to convey the meaning of phosphates to our patients, given that the word “phosphate” does not exist in the local language. We suspect that this is a problem in many other African countries. We usually explain that phosphates are substances in the food that accumulate in patients with renal failure but not in individuals with normal renal function. Its link with calcium is also explained. We then go on to highlight “damages” the calcium-phosphate imbalance can cause such as increased fractures, “hardening” of blood vessels and death. The association between hyperphosphatemia and pruritus is also stressed which often helps them to recognize the need for phosphate restriction. Almost all inorganic phosphates in processed foods is absorbed by humans while animal-based phosphorus is 40–60 % absorbable [19]. Plant-based organic phosphorus is even less absorbable in the human gut due to associated phytates. Advice on dietary phosphate restriction is done in accordance to these variabilities and thus restricts the use of food additives and also limit high organic phosphorus sources. We limit dairy products such as milk (*Maas*, *Amazi*), milk products (custard, cheese), ice-cream and cheese, high phosphate meats and substitutes such as eggs, pilchards, bacon, and sardines. With regards to high phosphate containing meats, we advise our patients to exchange these for low phosphate meats such as *bobotie*, lamb, chicken, mince, or fish (e.g. Cape snoek) (Table 3). We also encourage the inclusion of plant based proteins such as beans. Beyond phosphate restriction in diet, we educate our patients on the need to comply with the prescription of phosphate binders and also on the correct use of these binders i.e. chewing the binders just before or with meals.

### Approaches targeted to restrict sodium intake

The role of high salt intake in promoting fluid retention and contributing to poor blood pressure control is explained and reiterated at all clinic visits, or at dialysis sessions. In adapting the recommended daily sodium intake of 80–100 mmol/day in our setting, we employ a number of measures [12]. We emphasize that limiting salt intake requires more than taking salt off the dinner table but that it also includes not adding salt or salt alternatives such as Aromat®, onion salt, celery salt, garlic salt, meat tenderizers, or stock cubes to food during the cooking process. We advocate for alternative flavorings such as pepper, curry, chili, vinegar, onions, peppers, garlic, ginger, rosemary and lemon juice, and avoiding added salt to prepared meals (Additional file 1: Table S1). We additionally advice the avoidance of processed and/or canned foods (which are high in sodium) e.g. baked beans, processed meats (viennas, polony, bacon, ham, *biltong,* dried sausage, and dried fish –“*bokkoms*”), savory biscuits, chips (crisps and slap chips, also known as potato fries), instant soups, and sauces such as tomato sauce, chutney or Worcestershire sauce. Healthy snack alternatives offered include Provitas® rice cakes, unsalted nuts and popcorn.

### Approaches used to ensure fluid balance

Patients are made aware of the role of the kidneys in water regulation and how impaired kidney function leads to fluid retention. We create awareness on the symptoms of fluid retention (pedal edema, shortness of breath and an increasing body weight) and emphasize the need to balance fluid intake with urine output. What contributes to daily fluid intake is a common area of confusion among patients due to the misconception that only fluids such as water, coffee, tea, or soda contribute to daily fluid intake. Other sources of fluid intake (soups, sauces, gravies, yoghurt, and custard) are also pointed out. Patients are given practical solutions on managing thirst and fluid intake such as the need to evenly distribute fluid intake throughout the day, sucking on ice chips (while remembering to calculate these as part of overall fluid allowed), sipping rather than gulping down liquids, and managing body temperature depending on weather conditions. On hot summer days they may keep cool by wiping necks or faces with a damp cloth or sitting in shaded areas while during winter they should avoid the intake of hot liquids for the sole purpose of providing body heat. The relationship between salt intake and overall fluid homeostasis is also highlighted.

### Dietary approaches to potassium restriction

Our patients are educated on the consequences of potassium retention in CKD. In the SARE list, the potassium content in fruits and vegetables are divided into low, moderate, and high potassium-containing foods (Additional files 2 and 3: Tables S2 and S3) [18]. We use this as a guide in counselling our patients on how to choose their daily vegetable and fruit portions from the low or moderate potassium groups. For patients on continuous ambulatory peritoneal dialysis (CAPD), items from the high potassium group are permissible due to a relatively higher potassium allowance. For vegetables with high potassium content, patients are taught to leach away potassium by soaking vegetables in water and discarding the water before cooking, or boiling the vegetables in large amounts of water and discarding the broth. Starches such as potato and sweet potato are high in potassium and patients are counselled to avoid these as well as high potassium-containing fruits, e.g. citruses and bananas. Portion sizes in relation to overall potassium balance are emphasized. Other limited items include coffee, dried fruit, fruit juice, milk and dairy products like fruit yogurt, and chocolates.

### Approaches for carbohydrates, sugars and fat intake in CKD

Generally, the energy intake of CKD patients is recommended to be sufficient enough to maintain a neutral nitrogen balance [20, 21]. The recommended daily energy allowance among CKD-ND and CKD-5D patients is at least 30 kcal/kg (30 – 35kCal/kg) of ideal body weight [12, 13, 22]. Given the socio-economic constraints our patients face, it is inevitable that carbohydrates such as potatoes, bread and maize based meals will make up a substantial part of their diets, as these are relatively cheap. Our approach is therefore to educate on the need for adequate proportioning of carbohydrates and other food groups in a balanced diet. For our non-diabetic CKD patients we allow the intake of simple carbohydrates, such as jams, jelly babies and boiled sweets as can be allowed within daily energy limits. We however discourage the intake of sugary, fizzy drinks. Patients are also made aware of hidden sugars, such as those found in sauces. In CAPD patients, we ensure that the energy from the dextrose in the dialysate is factored into daily requirements and intake as highlighted in clinical practice guidelines [12, 13, 23].

In addressing appropriate fat intake, we discourage the intake of “hidden fats” in foods such as pies, *rotis*, *vetkoek*, doughnuts, *samoosas, bhajias*, coffee creamers and mayonnaise. We also recommend cooking methods such as boiling, baking, grilling or stewing rather than deep frying in an attempt to reduce the fat content of meals. Intake of low-fat dairy products, spreading butter or margarine thinly on bread is some other healthy options we encourage.

### Promotion of healthy habits

Patients are involved as much as possible in their meal planning and preparation as this leads to increased compliance. We encourage that meals be home-made and not bought from restaurants or food vendors. This ensures that a patient is in control of the ingredients used in making the meals and the methods employed in meal preparation. We discourage indiscriminate snacking (including snacking during dialysis sessions) while encouraging exercise and healthy sleeping patterns; these ultimately have an influence on energy levels, dietary intake and mental and physical health.

## Conclusions

While there are broad concepts involved in the nutritional management of CKD patients, the adaptation of these to local settings (giving varying racial and cultural nutritional practices and diets) guarantees a more tailored approach in the nutritional care of CKD patients. In multi-racial and multi-ethnic South Africa this strategy has proven acceptable to, and practicable among, our patients. The South African Renal Exchange list is currently a useful tool that has been adapted for different population groups in South Africa. Various challenges, driven mostly by poverty and illiteracy, however threaten a universally successful nutritional management plan among all our CKD patients.

## Abbreviations

CAPD, continuous ambulatory peritoneal dialysis; CKD, Chronic kidney disease; ESRD, end-stage renal disease; K/DOQI, kidney diseases outcomes quality initiative; KDIGO, Kidney Diseases Improving Global Outcomes; NKF, National Kidney Foundation; SARE, South African Renal exchange; SSA, Sub-Saharan Africa

## Additional files

Additional file 1: Table S1. Hints for flavoring food without addition of salt. (DOCX 12 kb) Additional file 2: Table S2. Common South African vegetables and standardized portions according to potassium content. (DOCX 17 kb) Additional file 3: Table S3. Standard portion sizes of South African fruits by potassium content. (DOCX 15 kb)
