# Supplementary material for: A practical approach to the nutritional management of chronic kidney disease patients in Cape Town, South Africa
Source: BMC Nephrol. 2016 Jul 8;17:68. doi: 10.1186/s12882-016-0297-4 (PMC4939026; doi:10.1186/s12882-016-0297-4)
Supplement: Additional file 1: Table S1. — Hints for flavoring food without addition of salt. (DOCX 12 kb) [file 12882_2016_297_MOESM1_ESM.docx]

Supplementary Table 1: Hints for flavoring food without addition of salt

| **Food** | **Recommended flavoring** |
| --- | --- |
| Beef | Dry mustard powder, bay leaves, thyme, sage, garlic, green pepper, onion, marjoram, paprika, apricot jam, pepper, oreganum |
| Mutton | Rosemary, ginger, pimento, garlic, brown sugar, pepper |
| Chicken | Lemon juice, parsley, paprika, thyme, green pepper and onion, garlic, bay leaves and black pepper, curry powder and apricot jam |
| Pork | Apple, applesauce, garlic, onion, sage, cloves, coriander, dry mustard powder, pepper |
| Fish | Bay leaves, lemon juice, oreganum, parsley, dry mustard powder, garlic and onion, pepper, ginger |
| Rice | Chopped spring onions, chopped green pepper, saffron, chopped parsley, melted margarine and garlic |
| Potatoes | Chopped spring onions, Chopped green pepper, chopped parsley, melted margarine and cream |
| Vegetables | Sweet: cinnamon and sugar (not for diabetics)  Salty: Pepper, garlic, onion, lemon juice, paprika, curry powder. |
