# Supplementary material for: A practical approach to the nutritional management of chronic kidney disease patients in Cape Town, South Africa
Source: BMC Nephrol. 2016 Jul 8;17:68. doi: 10.1186/s12882-016-0297-4 (PMC4939026; doi:10.1186/s12882-016-0297-4)
Supplement: Additional file 2: Table S2. — Common South African vegetables and standardized portions according to potassium content. (DOCX 17 kb) [file 12882_2016_297_MOESM2_ESM.docx]

Supplementary Table 2: Common South African vegetables and standardized portions** according to potassium content

| **Low Potassium** | | **Moderate Potassium** | | **High Potassium** | |
| --- | --- | --- | --- | --- | --- |
| **Item** | **Portion size** | **Item** | **Portion size** | **Item** | **Portion size** |
| Broccoli, cooked | ½ cup / 75g | Asparagus, cooked | ½ cup / 90g | Marog, cooked | 50g |
| Cabbage, cooked | ½ cup / 70g | Beetroot, cooked | 50g / 2 medium | Mixed vegetables, canned | ½ cup / 140g |
| Cabbage, raw | ½ cup / 40g | *Brinjal*, cooked with skin | ½ cup / 90g | Mushrooms, cooked, whole | ½ cup / 90g |
| Coleslaw | ½ cup / 50g | Brussel sprouts, cooked | ½ cup / 80g | Pumpkin, gem | ½ large / 90g |
| Lettuce, shredded | ½ cup / 40g | Carrots, cooked | ½ cup / 80g | Pumpkin, butternut | ½ cup / 105g |
| Mixed vegetables (carrot, corn, peas, beans) | ½ cup / 75g | Carrot salad, pineapple and orange juice | ½ cup / 65g | Tomato and onion stew, with sugar | 1 heaped ladle spoon / 75g |
| Onion, cooked | 50g / 1 med / 2 small | Carrots, raw | ½ cup / 50g |  |  |
| Peas, cooked | 50g / 2 heaped tablespoons | Cauliflower, cooked | ½ cup / 80g |  |  |
| Pumpkin, summer, marrow | ½ cup / 110g | Green beans, cooked | ½ cup / 65g |  |  |
| Sambal, tomato and onion | 50g | Mushroom, raw | ½ cup / 45g |  |  |
| Sweetcorn, cream style, canned | ¼ cup / 65g | Pumpkin, patty pan | 100g / 3 heaped tablespoons |  |  |
| Sweetcorn, whole kernel, canned | ¼ cup / 65g | Pumpkin, winter, white or hubbard | ½ cup / 105g |  |  |
| Tomato sauce | 1 level tablespoon / 25g | Spinach, cooked | ½ cup / 90g |  |  |
| Cucumber | 5 medium slices | Sweetcorn, cooked | ¼ cup / 65g |  |  |
| Pepper | 4 medium slices | Tomato, raw | 80g / 1 small |  |  |
|  |  | *Waterblommetjies*, fresh, boiled | 100g |  |  |

** Patients are allowed 2 to 4 portions depending on prescription
