# Supplementary material for: A practical approach to the nutritional management of chronic kidney disease patients in Cape Town, South Africa
Source: BMC Nephrol. 2016 Jul 8;17:68. doi: 10.1186/s12882-016-0297-4 (PMC4939026; doi:10.1186/s12882-016-0297-4)
Supplement: Additional file 3: Table S3. — Standard portion sizes of South African fruits by potassium content. (DOCX 15 kb) [file 12882_2016_297_MOESM3_ESM.docx]

Supplementary Table 3. Standard portion sizes of South African fruits by potassium content**

| **Low Potassium** | | **Moderate Potassium** | | **High Potassium** | |
| --- | --- | --- | --- | --- | --- |
| **Item** | **Portion size** | **Item** | **Portion size** | **Item** | **Portion size** |
| Apple | 100g / 1 small | Apricot | 50g / 2 small | Avocado | 40g / quarter |
| Cherry | 50g / 2 heaped dessertspoons | Grapefruit | Half | Fig | 100g / 2 large |
| Granadilla | 20g / 1 medium | Guava | 50g / 1 small | Grapes | 100g / 1small bunch |
| Litchi | 50g / 6 litchis | Melon, green-white | 60g / 3cm wedge | Kiwi | 100g / 1 fruit |
| Pear | 50g / ½ small | Naartjie | 100g /1med to large | Melon, yellow flesh | 60g / 3cm wedge |
| Pineapple | 40g / 2 med slices | Pawpaw | 100g / 4 heaped tablespoons | Orange | 120g / 1 small |
| Plum | 50g / 1 med | Gooseberry | 10 berries | Peach | 100g / 1 small |
| Strawberry | 100g / 8 med | Mango | Quarter | Banana | 50g / 1 small |
| Lemon | ½ medium |  |  | Watermelon | 100g / ½ wedge |
| Kumquats | 6 medium |  |  |  |  |

** Most patients will be allowed 2 to 3 portions depending on prescription
